# Quality of care for dual eligible beneficiaries in the oncology care model

**DOI:** 10.1002/cam4.70009

**Published:** 2024-07-18

**Authors:** Xinyu Liang, Ziwei Zhu, Kassem Faraj, Vahakn B. Shahinian, Brent K. Hollenbeck, Lindsey A. Herrel

**Affiliations:** ^1^ Ross School of Business University of Michigan Ann Arbor Michigan USA; ^2^ Department of Urology University of Michigan Ann Arbor Michigan USA; ^3^ Institute for Healthcare Policy and Innovation, University of Michigan Ann Arbor Michigan USA

**Keywords:** clinical management, medical oncology, QOL, quality of Life

## Abstract

**Introduction:**

Dual eligible beneficiaries are a vulnerable population who often experience inferior access to care and outcomes compared to non‐dual eligible beneficiaries. The Oncology Care Model (OCM) is an alternative payment model that aims to improve coordination and quality of care in beneficiaries receiving chemotherapy and thus may improve care for dual eligible beneficiaries with cancer.

**Methods:**

We used 100% Medicare claims data from 2014 through 2019 and included beneficiaries with bladder, breast, esophageal, colorectal, kidney, lung, pancreatic, or prostate cancer receiving chemotherapy. We constructed multivariable difference‐in‐differences regression models to evaluate the effect of OCM participation on healthcare utilization and quality of care at the end‐of‐life among dual eligible beneficiaries. We also compared healthcare utilization and quality of care outcomes to non‐dual eligible beneficiaries.

**Results:**

We identified 3,043,944 episodes of care among 1,260,892 unique Medicare beneficiaries. Ten percent of all beneficiaries (*n* = 126,758) were dual eligible and 64,087 (22%) of episodes among dual eligible patients were in an OCM participating practice. We noted no effect of OCM participation on healthcare utilization or end‐of‐life quality of care for dual eligible beneficiaries. However, we observed higher rates of hospitalization, emergency department visits, intensive care unit stays, and a lower number of office visits among dual eligible beneficiaries compared to non‐dual eligible beneficiaries.

**Conclusions:**

Participation in OCM was not associated with improvements in quality of care or healthcare utilization for dual eligible beneficiaries. Dual eligible beneficiaries experience lower quality of care across several measures compared to non‐dual eligible beneficiaries. Focused policies and incentives may be necessary to address disparities within emerging health reforms.

## Graphical abstract

Participation in the Oncology Care Model was not associated with improvements in quality or utilization for dual eligible beneficiaries. Dual eligible beneficiaries continue to experience lower quality across several utilization measures compared to non‐dual eligible beneficiaries.

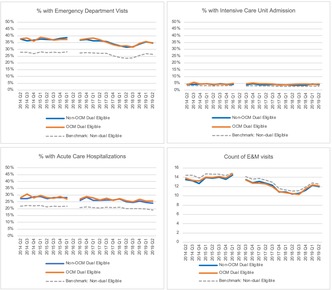

## 1 INTRODUCTION

As part of the effort to incentivize improvements in the quality of healthcare while reducing costs, the Centers for Medicare and Medicaid Services (CMS) Innovation Center developed the Oncology Care Model (OCM) in 2016. OCM is the first cancer‐focused payment model and is designed to improve healthcare delivery over 6‐month episodes of care for beneficiaries receiving chemotherapy.^1^ OCM is designed to incentivize coordinated, high quality care for beneficiaries treated in participating practices through a care management fee intended to support care navigation, adherence to guideline concordant care, and improved patient experience. Individual practices elected to participate in OCM through an application process with CMS. Participation is OCM at the practice level is voluntary. Participating practices are Medicare enrolled, cover urban, suburban, and rural areas across the nation, and range in size from solo oncologists to practices with hundreds of providers. All patients receiving chemotherapy within an OCM participating practice are included in the financial and performance evaluation. Early studies evaluating the impact of OCM have demonstrated declines in intensive care unit (ICU) admissions and emergency department use among participating beneficiaries and modest savings among certain groups.^2^, ^3^ Without specific attention to socioeconomically disadvantaged populations, such a dual eligible beneficiaries, it is unclear what impact OCM will have for these patients.

Dual eligible beneficiaries, those who simultaneously qualify for Medicare due to age or disability and Medicaid due to low income, account for about 20% of the Medicare population, but 34% of Medicare spending.^4^ They represent a uniquely vulnerable population, as more than 60% live below the federal poverty line, they are often from historically marginalized racial groups, with multiple comorbid conditions, and have a higher incidence of nearly all types of cancers.^5^ Dual eligible beneficiaries often experience lower overall quality of care, including cancer care and end‐of‐life care,^6^, ^7^ and represent an important population to monitor as federal health policy changes focused on improving the quality of care and reducing costs may offer opportunities to mitigate these disparities, however, could potentially create wider gaps in care for this socioeconomically disadvantaged group of patients. With extra resources focused on delivering highly coordinated, guideline concordant care, OCM may have the ability to improve care for dual eligible beneficiaries.

In this context, we used Medicare claims data to evaluate if OCM improved the quality of cancer care delivered to dual eligible beneficiaries. Though many of the disparities between this cohort and the general population exist outside of the healthcare setting, the focus on care coordination and high‐quality care that OCM provides may facilitate the additional steps towards equitable care in this population. This focus on quality of care within OCM and additional financial resources provided within the OCM model may also enable the delivery of higher quality of care at the end‐of‐life for patients with cancer, often a time period with high utilization and spending, particularly among dual eligible beneficiaries. Findings from this study will provide patients, physicians, and policymakers with information on the real‐world impact of national payment reforms on improving healthcare quality in vulnerable populations.

## 2 METHODS

### 2.1 Data sources

We used 100% cancer claims from Medicare files from 2014 through 2019 to perform our analyses.^8^ Within Medicare data, we specifically included and analyzed claims from the Medicare Provider Analysis and Review (MedPAR), Carrier, Outpatient, Hospice, Home Health Agency, and Part D Event files. These files were used to identify patient cohorts, demographic information, dual eligibility, and the occurrence of our outcomes of interest.

### 2.2 Identification of study sample and creation of OCM episodes

Our study population included Medicare beneficiaries aged 66–99 years receiving chemotherapy for a common solid organ malignancy who would be eligible for OCM participation. Our study period was 2014 through 2019. We first identified patients using an ICD9 or ICD10 diagnosis code for bladder, breast, esophageal, colorectal, kidney, lung, pancreatic, or prostate cancer from the second quarter of 2014 through the second quarter of 2019. Claims prior to diagnosis were used to identify comorbid conditions for risk adjustment. We excluded diagnoses after the second quarter of 2019 to allow complete claims in each episode. To ensure complete data for the cohort, we excluded individuals who did not have continuous enrollment in Medicare Parts A and B during the study period. We defined OCM episodes within this population using the methodology outlined by CMS.^1^ Briefly, we identified the first chemotherapy claim with a corresponding cancer diagnosis code in Part B (i.e., Carrier or Outpatient) or Part D claims, and this was the first month of the episode, which extended for six calendar months. We used OCM methodology and the provided list of chemotherapy codes downloaded from the CMS OCM website.^9^ If chemotherapy was ongoing, subsequent 6‐month episodes were generated. All claims for any diagnosis in the 6‐month episode were included consistent with OCM‐defined methodology. Cancer type for each episode was determined based on the plurality of E&M visit diagnosis codes. A washout period from February through June 2016, was implemented to account for OCM initiation on July 1, 2016, the time point at which 6‐month episodes began in OCM. This washout period allowed us to create parallel 6‐month episodes among the non‐OCM participating practices using the same rubric as creating OCM episodes.

Dual eligibility was defined at the episode level using monthly dual eligibility indicators in the Medicare Denominator File. Consistent with our prior work, as well as the work of MedPAC and MacPAC, episodes were flagged as dual eligible if any single monthly indicator was turned on.^10^ Finally, we identified patients who died during an episode of care and examined the quality of end‐of‐life care among this group of patients.

### 2.3 Identifying oncology care model providers

Once the patient's claims for chemotherapy and cancer were identified, we assigned each episode to a primary oncology provider/practice via the tax identification number (TIN) using a plurality rule consistent with CMS‐defined methodology. The TIN was then designated as participating in OCM or non‐participating using the presence of a non‐denied OCM monthly enhanced oncology services payment (i.e., the monthly CPT code for the care management fee billed exclusively during OCM participation, G9678) from the Part B claims.

### 2.4 Defining quality measures and outcomes

We defined and examined quality outcomes related to overall utilization within OCM and separately examined utilization of care at the end‐of‐life for those experiencing a cancer related death within an episode of care. We evaluated each outcome at the episode level using claims‐based measures. We examined several 6‐month episode overall utilization measures related to quality, including the percentage of episodes with acute care hospitalizations, emergency department (ED) visits, and ICU stays, as well as the number of evaluation and management (E&M) visits.

For the subgroup of patients who experienced a cancer related death within an episode of care, we examined seven end‐of‐life quality measures, including >1 hospitalization in the last 30 days of life, ICU admissions in the last 30 days of life, >1 ED visit in the last 30 days of life, receipt of chemotherapy in the last 14 days of life, death in an acute care setting, patients not enrolled in hospice at the time of death, and patients with less than 3 days in hospice among those who utilized hospice care.

### 2.5 Statistical analysis

We first examined the characteristics of our study population at the episode level, including both dual eligible beneficiaries and non‐dual eligible beneficiaries. We then compared the episode‐level characteristics of dual eligible beneficiaries whose episodes were attributed to OCM participating practices versus nonparticipating practices using *t*‐tests and chi‐squared tests.

Next, we constructed two separate difference‐in‐differences (DiD) analyses, the first model estimated the effect of OCM on quality outcomes among dual eligible beneficiaries only. To place our findings for dual eligible beneficiaries in the context of the overall quality of care that non‐dual eligible beneficiaries receive, we created a second model to estimate the quality of care that dual eligible beneficiaries experience compared to non‐dual eligible beneficiaries, an analysis that included all beneficiaries. These results of both models were overlaid on the same graph with the first analysis among only dual eligible patients in OCM and non‐OCM practice depicted in orange and blue lines respectively and a gray dashed line depicting the rates for non‐dual eligible beneficiaries. Models were constructed with time points at a quarterly level to alleviate the concern of potential time‐varying treatment effects. Using the DiD framework, we constructed multivariable linear models for continuous outcome variables, such as the number visits, as well as logistic regression models for binary variables, such as whether there are ED visits during the episode. We adjusted the DiD estimates for practice‐level characteristics (e.g., number of beneficiaries in practice receiving chemotherapy) and episode‐level factors, including beneficiary age, sex, geographical location (rural vs. urban areas), cancer type (to account for differences in intensity of care and other factors related to specific cancer types), presence of lower intensity cancer episode (i.e., certain breast, bladder, and prostate cancer episodes), hierarchical condition category count (HCC), and presence of surgery during the episode.^1^, ^3^ The standard errors of our model are clustered at the practice level to adjust for correlation between the episodes within a practice (i.e., TIN level). We tested the parallel trends assumption during the baseline period to ensure the suitability of using the DiD approach and adopted a dynamic approach by interacting the OCM participation indicator with quarter dummies to examine how the OCM impact changes over time. Our results suggest that the parallel assumption holds for our analyses. All statistical analyses were performed using SAS (version 9.4, SAS Institute Inc, Cary, North Carolina) and Stata statistical software packages (version 16; StataCorp, College Station, Texas). This study was deemed exempt by our institutional review board.

## 3 RESULTS

We identified 3,043,944 6‐month episodes for 1,260,892 unique Medicare beneficiaries between 2014 and 2019. Among these, 288,843 episodes were for 126,758 (10%) dual eligible beneficiaries and 2,755,099 episodes were for 1,134,134 (90%) non‐dual eligible beneficiaries. Among all episodes for dual eligible beneficiaries, 64,087 (22%) episodes were attributed to one of 195 OCM participating practices. The remaining 224,756 (78%) episodes were attributed to 4513 nonparticipating practices.

Table 1 displays episode characteristics stratified by dual eligibility. Compared to episodes for non‐dual eligible beneficiaries, episodes for dual eligible beneficiaries have a higher prevalence of comorbid conditions using Hierarchical Condition Categories (HCC) counts and a higher unadjusted incidence of death during an episode. We observed significant differences in the racial and sex distribution between dual eligible and non‐dual eligible beneficiaries. Episodes for dual eligible beneficiaries consist of fewer White (55% vs. 88%, *p* < 0.001) and male patients (38% vs. 46%, *p* < 0.001) than non‐dual beneficiaries.

**TABLE 1:** Patient characteristics by dual‐eligible versus non‐dual‐eligible (2014–2019).

Mean (SD)	Non‐dual	Dual	*p*‐value
No. of episodes	2,755,099	288,843	
No. of beneficiaries	1,134,134	126,758	
Age	76.1 (6.6)	76.2 (7.0)	
Sex			
Male	46%	38%	<0.001
Female	54%	62%	
Urban/rural			
Urban	77%	74%	<0.001
Rural	23%	26%	
Race and ethnicity			
Non‐Hispanic White	88%	55%	<0.001
Non‐Hispanic Black	6%	17%	
Hispanic	2%	16%	
Other^a^	3%	12%	
Cancer type			
Bladder	4%	3%	<0.001
Breast	42%	47%	
Esophagus	1%	1%	
Intestine	6%	7%	
Kidney	2%	2%	
Lung	12%	15%	
Pancreatic	3%	2%	
Prostate	29%	23%	
Hierarchical Condition Category count^b^			
0	36%	26%	<0.001
1	28%	27%	
2	17%	19%	
3	9%	12%	
4 to 5	7%	11%	
6 or more	3%	5%	
Lower‐risk^c^ episodes	25%	29%	<0.001
Death during episode	12%	15%	<0.001
Surgery during episode	4%	4%	<0.001

^a^ Other includes Asian/Pacific Islander, American Indian/Alaska Native, and other race.

^b^ Consistent with OCM methodology utilizing Hierarchical Condition Category count (Oncology Care Model Performance Periods 1 and 2 Payment Methodology (PDF) https://innovation.cms.gov/files/x/ocm‐cancercodelists.pdf pp. 75–76 Comorbidities section).

^c^ Lower‐risk episodes include low‐risk breast cancer (breast cancer episodes with hormonal therapy only), low‐intensity prostate cancer (prostate cancer episodes with hormonal therapy other than enzalutamide, abiraterone, and apalutamide), and low‐intensity bladder cancer (bladder cancer episodes treated with intravascular therapy only). All other episodes are considered higher‐risk episodes.

Table 2 shows episode characteristics by OCM participation for dual eligible beneficiaries. The distribution of cancer types differs for OCM and non‐OCM episodes among dual eligible beneficiaries. There were minimal differences between dual eligible beneficiaries who participated in OCM according to race (55.4% vs. 54.6% White in OCM vs. non‐OCM), ethnicity (17% vs. 16% Hispanic ethnicity in OCM vs. non‐OCM), and HCC comorbidity count compared to those dual eligible beneficiaries not participating in OCM. OCM episodes were more often for a primary diagnosis of breast cancer (51% vs. 45%, *p* < 0.001) compared to non‐OCM episodes of care. Lower‐risk cancer episodes made up a larger proportion of episodes for dual eligible beneficiaries participating in OCM compared to those not participating (31% vs. 28%, *p* < 0.001). There was a slightly higher unadjusted percentage of deaths during OCM episodes compared to non‐OCM episodes (16% vs. 14%, *p* < 0.001).

**TABLE 2:** Patient characteristics by OCM versus Non‐OCM (2014–2019).

Mean (SD)	Non‐OCM	OCM	*p*‐value
No. of episodes	224,756	64,087	
Age	76.2 (7.0)	76.3 (7.0)	
Sex			
Male	39%	33%	<0.001
Female	61%	67%	
Urban/rural			
Urban	73%	78%	<0.001
Rural	27%	22%	
Race and ethnicity			
Non‐Hispanic White	55%	55%	<0.001
Non‐Hispanic Black	18%	17%	
Hispanic	16%	17%	
Other^a^	12%	11%	
Cancer type			
Bladder	3%	2%	<0.001
Breast	45%	51%	
Esophagus	1%	1%	
Intestine	7%	8%	
Kidney	2%	2%	
Lung	15%	16%	
Pancreatic	2%	2%	
Prostate	25%	18%	
Hierarchical Condition Category count^b^			
0	26%	25%	<0.001
1	27%	27%	
2	19%	19%	
3	12%	12%	
4 to 5	11%	12%	
6 or more	5%	5%	
Lower‐risk^c^ episodes	28%	31%	<0.001
Death during episode	14%	16%	<0.001
Surgery during episode	4%	4%	<0.001

^a^ Other includes Asian/Pacific Islander, American Indian/Alaska Native, and other race.

^b^ Consistent with OCM methodology utilizing Hierarchical Condition Category count (Oncology Care Model Performance Periods 1 and 2 Payment Methodology (PDF) https://innovation.cms.gov/files/x/ocm‐cancercodelists.pdf pp. 75–76 Comorbidities section).

^c^ Lower‐risk episodes include low‐risk breast cancer (breast cancer episodes with hormonal therapy only), low‐intensity prostate cancer (prostate cancer episodes with hormonal therapy other than enzalutamide, abiraterone, and apalutamide), and low‐intensity bladder cancer (bladder cancer episodes treated with intravascular therapy only). All other episodes are considered higher‐risk episodes.

**FIGURE 1:**
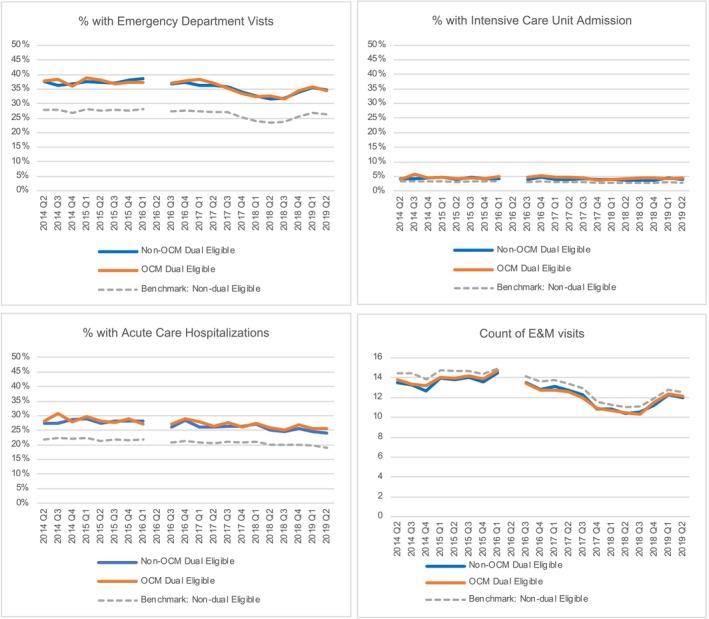
Multivariable difference‐in‐differences model results evaluating 6‐month episode utilization measures for dual eligible beneficiaries according to OCM participation. Percentage of episodes with ED visit, ICU stay, and acute care hospitalizations for dual eligible beneficiaries in OCM (orange) and not participating in OCM (blue) with non‐dual eligible beneficiary mean for reference (gray dashed line) by quarter. Count of E&M visits per 6‐month episode by quarter. No significant differences between dual eligible beneficiaries in OCM versus not participating in OCM (DiD estimator *p* > 0.05).

Figure 1 demonstrates the results of our multivariable DiD models evaluating time trends of risk‐adjusted quality outcomes of OCM episodes (orange line) and non‐OCM episodes (blue line) among dual eligible beneficiaries, with non‐dual eligible beneficiaries as the benchmark (gray dashed line). First, we did not observe any significant differences in these outcomes for dual eligible beneficiaries participating in OCM compared to their non‐OCM participating counterparts (no difference in change over time between blue and orange lines, DiD estimator *p* > 0.05). However, the gap between dual eligible beneficiaries (blue and orange lines) and non‐dual eligible beneficiaries (gray dashed lines) across quality measures is persistent and did not significantly change over time. Dual eligible beneficiaries have higher rates of acute care hospitalizations, emergency department visits, ICU admissions, and lower counts of E&M visits in comparison with non‐dual‐eligible beneficiaries (all *p* < 0.001 for dual eligible vs. non‐dual eligible).

**FIGURE 2:**
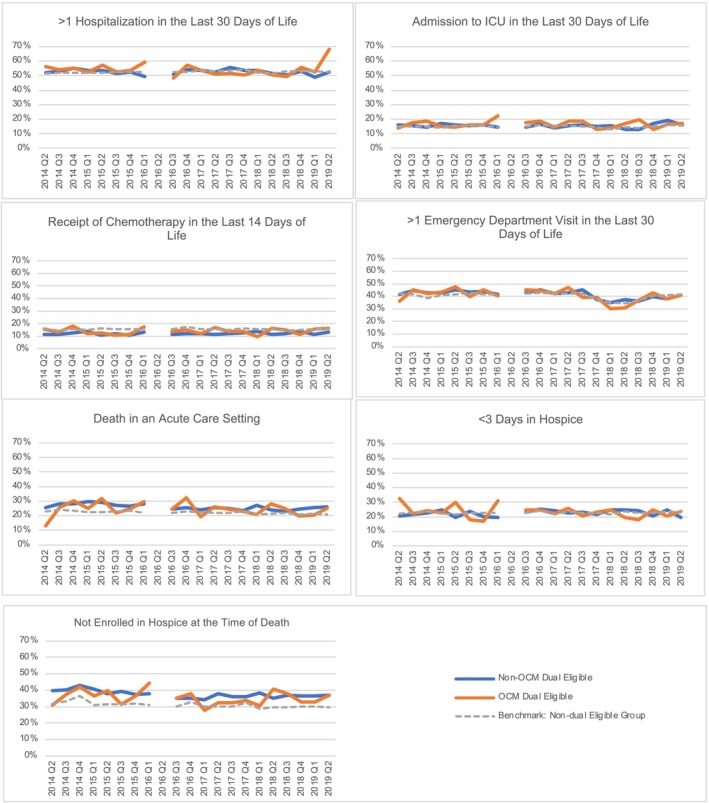
Multivariable difference‐in‐differences model results evaluating end‐of‐life quality measures for dual eligible beneficiaries according to OCM participation. Percentage of episodes where the beneficiary died and experienced adverse end‐of‐life outcomes for dual eligible beneficiaries in OCM (orange) and not participating in OCM (blue) with non‐dual eligible beneficiary mean for reference (gray dashed line) by quarter. No significant differences between dual eligible beneficiaries in OCM versus not participating in OCM (DiD estimator *p* > 0.05). Differences between dual eligible and non‐dual eligible were significant (*p* < 0.001 for death in acute care setting, enrollment in hospice at the time of death, and emergency department visits in the last 30 days).

Figure 2 shows the results of our multivariable DiD models evaluating risk‐adjusted outcomes of seven end‐of‐life quality measures over time among non‐dual eligible beneficiaries and dual eligible beneficiaries according to OCM status. We again noted no significant difference in any of the seven end‐of‐life quality measures for dual eligible beneficiaries participating in OCM compared to those not participating in OCM over time (DiD estimator *p* > 0.05). However, we again noted differences between dual eligible and non‐dual eligible beneficiaries with dual eligible beneficiaries demonstrating higher rates of death in an acute care setting, higher rates of >1 emergency department visit in the last 30 days of life, and lower rates of hospice utilization (*p* < 0.001).

## 4 DISCUSSION

Our study has two main findings. First, the quality of cancer care for dual eligible beneficiaries is not improved by participation in OCM despite OCM's emphasis on care coordination and delivering high quality cancer care. Second, disparities in the quality of cancer care persist between dual eligible beneficiaries and non‐dual eligible beneficiaries across several key measures of utilization of care and end‐of‐life care that are relevant for emerging health policies. This suggests that a sharper focus on improving health equity in future iterations of OCM will be necessary to elevate the quality of cancer care for dual eligible beneficiaries.

Recent literature evaluating the impact of OCM shows an overall modest impact of the program in terms of improving quality and controlling spending for patients with cancer. In particular, authors have noted small declines in ICU admissions and emergency room visits, but no impact on hospitalizations, 30 day readmissions, or total episode payments.^2^ More recently, Keating et al. demonstrated that in its first 3 years since implementation, OCM was associated with total payment reductions of $297 per episode, which was not sufficient to cover the monthly enhanced oncology services payments offered through OCM.^3^ Our work and others have shown that characteristics of the hospital where you receive care are associated with quality of care for dual eligible beneficiaries such as higher intensity utilization at the end of life for beneficiaries with cancer, and higher readmissions after hospitalization for medical conditions such as pneumonia and myocardial infarction.^11^, ^12^, ^13^ Despite evident benefits for some models of care, our study shows that OCM participation did not improve quality outcomes among dual eligible beneficiaries.

Our study has several limitations. First, our study utilizes Medicare claims which fail to capture certain patient‐level factors, such as cancer stage, patient treatment preferences, or OCM registry data. Despite this, claims data do provide detailed utilization and spending information and are the basis of OCM methodology. Second, our study compares episode‐level utilization and quality outcome trends, which may be impacted by potential selection biases inherent in the voluntary participation in the OCM payment program (e.g., high‐quality practices may be more inclined to opt‐into OCM and offer a better quality of care to cancer patients). This has been a variable aspect of value‐based programs with some being voluntary (e.g., Accountable Care Organizations) and others being mandatory (e.g., Comprehensive Care for Joint Replacement) and the role of voluntary participation continues to be evaluated at the programmatic level by the CMS. Last, our study evaluates the impact of OCM within a specific time frame (2014–2019) and for a specific set of solid organ malignancies, which may not fully capture the long‐term effects of the program. Our findings therefore may not be generalizable to patients with other solid organ malignancies or blood cancers. Additionally, our study evaluated only patients receiving chemotherapy and for our end‐of‐life measures, those who died from cancer during an episode and are not reflective of the entire scope of cancer care. The OCM evaluated in this study has ended as planned and a new version, the Enhancing Oncology Model, has rolled out.^14^ Building on lessons learned from the OCM, the new model focuses on a smaller number of cancers and provides a lower care management fee to help lower the overall cost of the program. The new model now requires social needs screening for patients and provides an additional care management fee for dual eligible beneficiaries suggesting a greater interest in the models ability to improve health equity.

Our findings have important implications for patients, physicians, and policymakers. First, there is ample room to improve the quality of cancer care for dual eligible beneficiaries and eliminate the disparity for this vulnerable population. However, our findings emphasize that broad policies lacking a specific focus on health equity, such as OCM, are ineffective in reducing existing disparities. While OCM was not specifically designed to close gaps in health equity, many hoped that the strong emphasis on quality of care combined with financial resources to focus on care coordination, patient navigation, and adherence to guideline concordant care, would improve quality of care for vulnerable patients who often experience fragmented care. Social drivers of health are known to impact health outcomes, and social risk factors such as food insecurity, housing instability, and lack of transportation among patients with cancer, particularly those who are dual eligible, are likely impact access to high quality care at a variety of levels, which inevitably exacerbate differences in overall outcomes. For policymakers, providing directed incentives and resources to address social health needs among vulnerable populations will be critical to improve the quality of care as health reforms are reshaped and as new efforts emerge. Practically, our current healthcare delivery system has insufficient resources to address the enormous burden of social risk factors that patients with cancer experience. The new Enhancing Oncology Model has some process measures and funds targeted specifically for dual eligible patients, but ongoing evaluations will be necessary to see if these go far enough to improve care. Building programs that specifically engage dual eligible patients will be critical for the CMS as dual eligible beneficiaries disproportionately account for adverse outcomes and increased spending among Medicare populations. By tailoring interventions and support for this specific patient population either at the federal, state, or local community level, we can learn ways to construct future programs equipped to address disparities and enhance the quality of cancer care for all.

## 5 CONCLUSIONS

Overall, OCM did not impact quality and utilization among dual eligible beneficiaries. Significant disparities in quality and utilization remain between dual eligible and non‐dual eligible beneficiaries receiving cancer treatment. It suggests that more targeted payment policies can be designed and implemented to reduce the healthcare quality gap between two dual and non‐dual eligible beneficiaries.

## AUTHOR CONTRIBUTIONS

**Xinyu Liang:** Conceptualization (equal); data curation (supporting); formal analysis (equal); investigation (equal); methodology (equal); validation (equal); visualization (equal); writing – original draft (equal); writing – review and editing (equal). **Ziwei Zhu:** Conceptualization (equal); data curation (lead); formal analysis (lead); investigation (equal); methodology (lead); software (equal); validation (equal); visualization (equal); writing – original draft (equal); writing – review and editing (equal). **Kassem Faraj:** Conceptualization (equal); investigation (equal); validation (equal); writing – review and editing (equal). **Vahakn B. Shahinian:** Conceptualization (supporting); investigation (supporting); methodology (supporting); supervision (supporting); writing – review and editing (supporting). **Brent K. Hollenbeck:** Conceptualization (supporting); funding acquisition (supporting); investigation (supporting); methodology (supporting); resources (supporting); supervision (supporting); validation (supporting); writing – review and editing (supporting). **Lindsey A. Herrel:** Conceptualization (lead); data curation (equal); formal analysis (supporting); funding acquisition (lead); investigation (lead); methodology (equal); project administration (lead); resources (equal); software (supporting); supervision (lead); validation (equal); visualization (equal); writing – original draft (lead); writing – review and editing (equal).

## CONFLICT OF INTEREST STATEMENT

The authors have no conflict of interest to declare.

## ETHICAL/IRB STATEMENT

This study was deemed exempt by the University of Michigan Institutional Review Board.

## Data Availability

The data that support the findings of this study are available from the Centers for Medicare and Medicaid Services (CMS). Restrictions apply to the availability of these data, which were used under a data use agreement for this study. Data are available from ResDAC.
